# Longitudinal tracking of axonal loss using diffusion magnetic resonance imaging in multiple sclerosis

**DOI:** 10.1093/braincomms/fcac065

**Published:** 2022-03-17

**Authors:** Frederique M. Boonstra, Meaghan Clough, Myrte Strik, Anneke van der Walt, Helmut Butzkueven, Owen B. White, Meng Law, Joanne Fielding, Scott C. Kolbe

**Affiliations:** 1 Department of Neuroscience, Central Clinical School, Monash University, Level 6, 99 Commercial Rd, Prahran 3005, Australia; 2 Department Radiology, Alfred Health, Prahran 3005, Australia; 3 Department of Medicine and Radiology, University of Melbourne, Parkville 3010, Australia

**Keywords:** multiple sclerosis, diffusion magnetic resonance imaging, axonal degeneration, longitudinal cohort study

## Abstract

Axonal loss in the CNS is a key driver of progressive neurological impairments in people with multiple sclerosis. Currently, there are no established methods for tracking axonal loss clinically. This study aimed to determine the sensitivity of longitudinal diffusion MRI-derived fibre-specific measures of axonal loss in people with multiple sclerosis. Fibre measures were derived from diffusion MRI acquired as part of a standard radiological MRI protocol and were compared (i) to establish measures of neuro-axonal degeneration: brain parenchymal fraction and retinal nerve fibre layer thickness and (ii) between different disease stages: clinically isolated syndrome and early/late relapsing–remitting multiple sclerosis. Retrospectively identified data from 59 people with multiple sclerosis (18 clinically isolated syndrome, 22 early and 19 late relapsing–remitting) who underwent diffusion MRI as part of their routine clinical monitoring were collated and analysed. Twenty-six patients had 1-year and 14 patients had a 2-year follow-up. Brain parenchymal fraction was calculated from 3D MRI scans, and fibre-specific measures were calculated from diffusion MRI using multi-tissue constrained spherical deconvolution. At each study visit, patients underwent optical coherence tomography to determine retinal nerve fibre layer thickness, and standard neurological assessment expanded the disability status scale. We found a significant annual fibre-specific neuro-axonal degeneration (mean ± SD = −3.49 ± 3.32%, *P* < 0.001) that was ∼7 times larger than the annual change of brain parenchymal fraction (−0.53 ± 0.95%, *P* < 0.001), and more than four times larger than annual retinal nerve fibre layer thinning (−0.75 ± 2.50% *P* = 0.036). Only fibre-specific measures showed a significant difference in annual degeneration between the disease stages (*P* = 0.029). Reduced brain parenchymal fraction, retinal nerve fibre layer thickness and fibre-specific measures were moderately related to higher expanded disability status scale (rho = −0.368, rho = −0.408 and rho = −0.365, respectively). Fibre-specific measures can be measured from data collected within a standard radiological multiple sclerosis study and are substantially more sensitive to longitudinal change compared with brain atrophy and retinal nerve fibre layer thinning.

## Teaser

This study investigated fibre-specific diffusion MRI in 40 clinically stable people with multiple sclerosis as a marker of progressive neuro-axonal loss. Kolbe et al. report significant changes in fibre-specific measures over only 18 months and significantly greater sensitivity compared with retinal nerve fibre loss and brain atrophy.

## Graphical Abstract

**Graphical Abstract:**
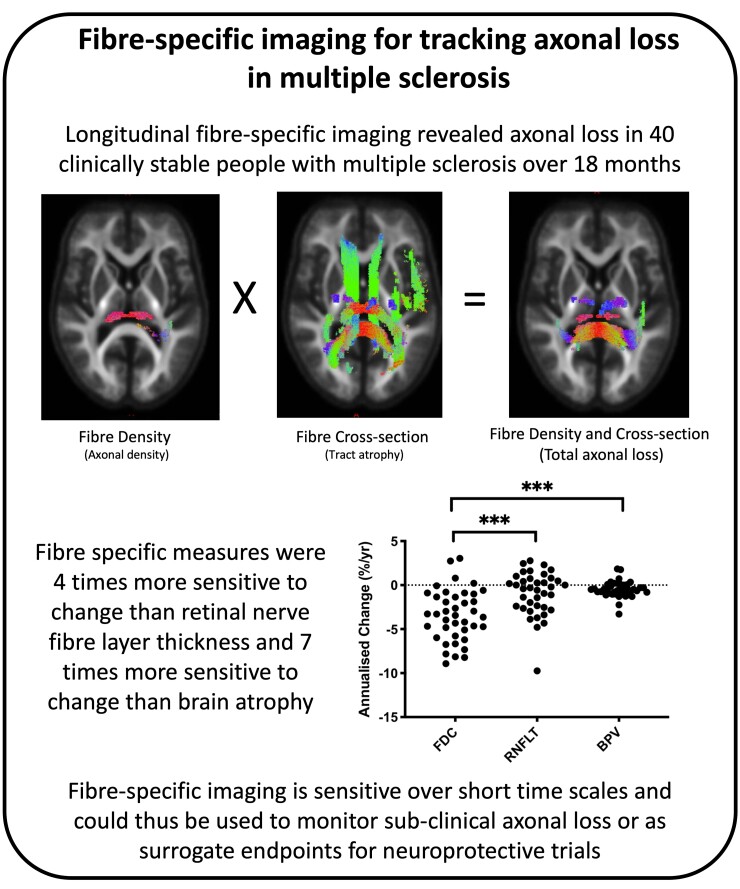

## Introduction

Irreversible neuro-axonal degeneration is a key pathological feature of multiple sclerosis and is likely to be the main pathological driver of long-term neurological progression.^1,2^ Detecting and monitoring axonal loss from the earliest stages of multiple sclerosis is therefore important for identifying patients at risk of more aggressive disease and poorer long-term clinical outcomes. Markers of axonal loss could also provide outcome measures for early-stage clinical trials of neuroprotective therapies, or as phenotypic markers for studies aiming to identify genetic variants associated with multiple sclerosis severity.

Several paraclinical imaging measures have been proposed as markers of neuro-axonal degeneration in multiple sclerosis, with brain atrophy the current gold standard.^3^ Brain atrophy is a consequence of axonal loss and can be measured on MRI. Importantly, brain atrophy is already present in the early stages of multiple sclerosis.^4^ Furthermore, the latest definition of no disease activity or ‘no evidence of disease activity’ (NEDA),^5^ includes brain volume loss of <0.4% alongside the three previous criteria of no relapses, active lesions and disability progression.^6^ However, brain atrophy measurement lacks sensitivity over clinically relevant time periods (∼1 year) and does not provide specific information regarding which brain regions and networks are exhibiting accelerated degeneration.^6–8^

Optical coherence tomography (OCT) is a retinal imaging technique that measures the thickness of retinal layers including the unmyelinated axons of the optic nerve (retinal nerve fibre layer; RNFL) and has been proposed to be a surrogate marker of brain atrophy and axonal loss in multiple sclerosis.^9–11^ A recent systematic review^12^ demonstrated the robustness and large effect size of the technique in identifying differences between the RNFL thickness in people with multiple sclerosis (pwMS) with and without optic neuritis compared with healthy controls. However, uncertainty remains regarding the utility of OCT as a marker of progression beyond the visual system. There has thus been significant interest in the development of neuroimaging-based markers that are sensitive and specific to neuro-axonal degeneration in brain regions with known functions and which allow better monitoring and clinical decision making regarding neurological decline in multiple sclerosis.

Diffusion MRI (dMRI) is sensitive to the molecular motion of water in tissue. Axons provide numerous aligned barriers to diffusion such that loss of axons leads to measurable increases in molecular diffusion perpendicular to the longitudinal axis of the axons.^13^ When diffusion is measured in numerous directions, a distribution of axonal fibre orientations can be calculated for each voxel to measure damage to specific fibre bundles. Recent technical developments have allowed for the calculation of fibre-specific measures of density and morphological changes commonly referred to as ‘fixel-based analysis’.^14^ First, by strongly weighting the diffusion signal to remove the more freely diffusing components in the extracellular space, intra-axonal water can be targeted specifically to obtain a measurement of axonal fibre density (FD) in specific white matter tracts.^15^ In addition, fibre cross section (FC) is a measure of tract atrophy calculated from the elastic transformation of an individual subject’s brain to an unbiased template. An estimate of total axonal count can thus be calculated as the multiplication of FD and FC (termed FD and cross section or FDC). Demonstrating the specificity of the technique in multiple sclerosis, previous studies have reported reduced FD and FC in the visual pathways of a pwMS with a history of acute optic neuritis^16^ and in sensorimotor networks in a pwMS with gait and balance disturbances.^17^ Despite the apparent strengths of fibre-specific imaging as an axonal marker, assumptions regarding the relationship between true axonal density and dMRI-measured FD are only valid for strong diffusion weighting (*b*≥3000 smm^−2^), where the signal from extracellular water is completely annulled. Given that most clinical dMRI sequences use weaker diffusion weighting (*b*≈1000 smm^−2^), accurate FD measurement requires sequence modifications and is thus generally not clinically acquired.

In this study, we examined the use of fibre-specific measures of axonal loss calculated from optimized dMRI data in a retrospective convenience sample collected during a routine clinical MRI follow-up. We aimed to determine the longitudinal sensitivity of fibre-specific measures and to compare fibre-specific measures: (i) with the degree of change in other proposed markers of neurodegeneration in multiple sclerosis (brain atrophy and RNFL thinning), and (ii) between different disease stages [clinically isolated syndrome (CIS), early/late relapsing–remitting multiple sclerosis (RRMS)]. We hypothesized that fibre-specific measures, as more accurate imaging measures of neuro-axonal degeneration, would be more sensitive to longitudinal change than more derivative measures such as brain atrophy or RNFL thinning. We also expected that fibre-specific measures would indicate progressive axonal loss at all disease stages consistent with the hypothesis that neuro-axonal loss commences early in the disease and is ongoing throughout the disease course.

## Methods

### Study subjects and clinical assessments

A convenience sample of 59 pwMS underwent clinical MRI sessions at the Royal Melbourne Hospital as part of their routine clinical care over the time period of December 2010–October 2014. pwMS were categorized into three groups based on clinical diagnosis^18^ and disease duration at baseline: (i) CIS (*n* = 18, 34.1 ± 7.5 years and 77.8% female), (ii) early RRMS (disease duration <5 years, *n* = 22, 38.6 ± 9.6 years and 86.6% female), and (iii) late RRMS (disease duration >5 years, *n* = 19, 49.2 ± 9.8 years and 94.7% female). Longitudinal data were available for 40 patients with serial MRI scans: 26 patients (7 CIS, 10 early, 9 late RRMS) with one follow-up MRI scan and 14 patients (5 CIS, 5 early, 4 late RRMS) with two follow-up MRI scans. The average follow-up time for patients with serial scans was 18 months irrespective of the number of scans. Four CIS patients converted to RRMS during follow-up. Only six patients (all RRMS phenotype) showed a single contrast-enhancing lesion at any time point during follow-up. With the exception of a single patient who displayed a single-step EDSS worsening (4 to 5), no patients experienced disability worsening during follow-up. At the time of the study, the majority of patients were on disease-modifying therapies with 37 on Fingolimod, four on Avonex/Betaferon/Copaxone, one on Natalizumab and seven on no DMT. Data were not available for the remaining 10 patients.

All patients underwent a standard neurological examination by a consultant neurologist (O.W.) for calculating the expanded disability status scale (EDSS). All data were retrospectively collated and analysed several years after collection, thus EDSS scores from four patients and RNFL thickness from three patients were unobtainable at the time of analysis as these patients moved to an alternate hospital outpatient clinic and data could not be obtained. All patients provided informed written consent for the use of their data for research purposes, and the study was approved by the Melbourne Health Human Research Ethics Committee.

### Optical coherence tomography

RNFL thickness was measured using either a Nidek Navis or Heidelberg Instruments Spectralis spectral-domain OCT systems. For all scans, the disc position was selected manually before each scan. No subjects required pupil dilation before scanning. Patients with multiple visits were always scanned using the same OCT system, and subsequent scans were positioned according to the original position to make a comparison between visits reliable. For patients with no evidence of optic nerve involvement, the average RNFL thickness of both eyes was used for statistical analyses. Thirteen pwMS had a clinical history of acute optic neuritis and a further five patients had an indication of sub-clinical optic nerve involvement (>5 µm interocular difference in RNFL thickness).^19^ For those participants, the RNFL thickness of only the unaffected eye was used.

### MRI acquisition

A standard-of-care radiological multiple sclerosis protocol was performed using a 3 T MRI system (Magnetom Trio, Siemens, Erlangen) with a 12-channel receiver coil. The clinical imaging protocol included a minimum of:

1. Sagittally acquired 3D fluid-attenuated inversion recovery (FLAIR): TR/TE/TI = 5000/350/1800 ms; FA = 120°; in-plain resolution = 0.5 × 0.5 mm^2^; slice thickness = 1 mm.
2. Axially acquired contiguous two-dimensional (2D) T_1_-weighted axial FLASH with and without single-dose gadolinium injection: TR/TE = 250/2.5 ms; FA = 70°; in-plain resolution = 0.42 × 0.42 mm^2^; slice thickness = 5 mm.
3. Axially acquired 2D spin-echo echo-planar diffusion-weighted imaging: TR/TE = 8600/120 ms; FA = 90°; in-plain resolution = 0.42 × 0.42 mm^2^; slice thickness = 5 mm; 1*non-diffusion and 30*directionally encoded diffusion-weighted images with gradient *b*-value = 3000 s/mm^2^.

Although not routinely acquired at the time of data collection for this study, for a sub-set of 24 patients, a single sagittally acquired 3D T_1_-weighted magnetization prepared - rapid gradient echo (MPRAGE) sequence was acquired and used to validate brain volumes calculated from FLAIR: TR/TE/TI = 2300/2.98/900 ms; FA = 9°; in-plain resolution = 1 × 1 mm^2^; slice thickness = 1 mm.

### Brain volume and lesion assessments

For each time point in each patient, gadolinium enhancing lesions were identified and their number recorded by an experienced rater (S.K.). Brain and lesion volumes were calculated from 3D FLAIR scans using a fully automated analysis pipeline. For each subject, a brain mask was calculated using the SPM12 segmentation algorithm. Due to inaccurate grey/white matter segmentation on FLAIR, grey and white matter volumes were not analysed separately, but added together to calculate total brain volume. The cerebrospinal fluid mask was added to the brain mask to make an intra-cranial mask for calculation of brain parenchymal fraction. Lesions were automatically segmented using the Lesion Prediction Algorithm contained within the Lesion Segmentation Toolbox for SPM12. Lesion masks were generated from LPA outputs and then used for inpainting the brain masks. From these masks the following volumes were calculated: intra-cranial volume, lesion volume, brain volume.

Brain volume was calculated from the sub-group of MPRAGE scans using an identical procedure to that used for FLAIR. For each subject, a brain mask was calculated using the SPM12 segmentation algorithm and total brain volume was calculated as the sum of grey and white matter volume. To validate FLAIR brain volumes, FLAIR and MPRAGE derived brain volume estimates were compared using intra-class correlation and Blandt-Altman plots (calculated using SPSS v26). Intra-class correlation was very high for comparisons between FLAIR and MPRAGE derived brain volumes (*R* = 0.994, 95% CI [0.987 to 0.997], Supplementary Fig. 1A). Blandt-Altman analysis showed that there was a systematic bias towards greater volumes derived from FLAIR (mean [SD] volume 1.170 L [0.118]) compared to MPRAGE (1.156 L [0.118]), yet there was no relationship between the subject-wise difference in volumes and the mean (Supplementary Fig. 1B).

### dMRI analysis

Diffusion MRI data were processed using the fixel-based analysis pipeline in MRtrix 3.0 (https://www.mrtrix.org/). A brief description of the pipeline is as follows and is presented diagrammatically in Supplementary Fig. 2.

1. Raw dMRI data were preprocessed to correct for noise,^20^ eddy currents and bias fields.^21^
2. For each brain voxel, the fibre orientation distribution (FOD) was calculated using constrained spherical deconvolution.^22^ The FOD is a 3D distribution model for axonal fibre directions in each image voxel with lobes reflecting the primary axes of orientation for each fibre bundle passing through a voxel.
3. An unbiased FOD template was created from a single dMRI data set for each subject, and all subjects’ data for all time points were non-linearly registered to the template^23^ and the log-Jacobian of the registration warp field was saved.
4. The FOD lobes were segmented to identify individual fibre bundles. This quantization of fibre bundles into fibre elements (fixels) is analogous to the quantization of 3D images into voxels, with individual voxels potentially containing more than one fixel if multiple intersecting fibre bundles intersect with that voxel. For each fixel, fibre-specific measures were calculated [FD, FC and FD corrected for FC (FDC)]. FD was derived by using the integral of the FOD lobule, and FC was estimated using the Jacobian of the warp field, and FDC equals FD×FC.
5. Whole-brain tractography was performed using the FOD template to calculate fixel-to-fixel connectivity.
6. For baseline analyses: mean FD, FC and FDC were calculated for the whole brain from all baseline scans and used for cross section statistical analyses.
7. For longitudinal analyses: fixels with progressive loss of FD, FC and FDC across the group were identified using mixed-effects general linear models (GLMs) (random factor: subject; covariates: time from first scan, age, sex).^24^*P*-values for the fixels, where a change in FD, FC and FDC correlated with time elapsed, were corrected multiple comparisons using cluster-based enhancement based on fixel-to-fixel connectivity. Mean FD, FC and FDC of significant fixels were calculated from all scans in all patients and used for longitudinal statistical analyses.

### Statistical analyses

Normality was tested for all variables. For analyses including the EDSS, we used non-parametric tests. For all other variables parametric tests were used; however, the FC was log-transformed when used in correlations. Differences in clinical and imaging variables between CIS, early RRMS and late RRMS were tested for using a one-way ANOVA for scale variables and a Pearson χ^2^ test for binary variables. Baseline correlations between imaging measures [brain parenchymal fraction (BPF), lesion fraction, RNFL thickness and whole-brain fibre-specific measures), demographics and disability (EDSS) was performed using Pearson’s correlation and Spearmen rank correlation. Annual change in imaging measures (BPF, lesion fraction, RNFL thickness and fibre-specific measures within significant tracts) was expressed as a percentage change between follow-up and baseline visits divided by the time between visits. One-sample *t*-tests were performed to determine whether the annual change scores were significantly different from zero. Correlation between the annual change of the different imaging measures was performed using Pearson correlation. Patients were stratified based on the annualized brain volume change threshold used for NEDA-4 criteria (>/<0.4% brain volume loss per annum).^6^ Differences in baseline and annual change measures between (>/<0.4% brain volume loss groups were tested for using independent *t*-test for scale variables and a Pearson χ^2^ test for binary variables. Finally, a one-sample *t*-test was performed to determine whether patients with >/<0.4% annual loss in BPF showed non-zero changes in fibre-specific measures. Owing to the exploratory nature of the study, no adjustment for multiple comparisons was made for analyses other than fixel-based image analyses.

### Data access and data availability

De-identified copies of raw and summary data for this study can be made available to qualified investigators upon reasonable request to the corresponding author.

## Results

### Baseline comparisons: all participants and between multiple sclerosis phenotypes

Table 1 shows baseline demographic, disease severity and imaging data from all patients and patient sub-groups. Age and disease duration were significantly different between the groups. Furthermore, there were significant group differences in BPF, lesion fraction and RNFL thickness. *Post hoc* analyses showed that this difference was only significant between CIS and late RRMS patients. We also found a significant group difference in whole-brain FDC, and *post hoc* analyses showed that the difference was between CIS and early RRMS. No significant differences were found for whole-brain FD and FC.

**Table 1:** Demographics of all multiple sclerosis patients and divided into the phenotypic sub-groups

*N*	All patients	CIS	Early RRMS	Late RRMS	*P*
	59	18	22	19	
Age (years), mean (SD)	40.6 (10.9)	34.1 (7.53)	38.6 (9.60)	49.2 (9.84)	***P* < 0.001**
Sex, female %	86.4	77.8	86.4	94.7	*P* = 0.322
Disease duration (years), mean (SD)	6.22 (7.49)	2.34 (4.16)	1.92 (1.36)	14.9 (6.75)	***P* < 0.001**
EDSS, median (IQR)	0.0 (0.0, 1.0)	0.0 (0.0, 0.0)	0.0 (0.0, 1.0)	0.0 (0.0, 1.5)	*P* = 0.202
BPF (%), mean (SD)	85.0 (4.79)	87.0 (4.09)	85.2 (4.16)	83.0 (5.44)	***P* = 0.036**
Lesion fraction (%), mean (SD)	0.49 (0.53)	0.25 (0.20)	0.48 (0.38)	0.74 (0.77)	***P* = 0.016**
RNFL thickness (μm), mean (SD)	97.3 (13.2)	103.9 (11.3)	97.7 (12.8)	90.9 (12.9)	***P* = 0.011**
FDC whole brain, mean (SD)	0.36 (0.03)	0.37 (0.03)	0.35 (0.03)	0.35 (0.03)	***P* = 0.040**
FD whole brain, mean (SD)	0.33 (0.01)	0.34 (0.01)	0.33 (0.01)	0.33 (0.02)	*P* = 0.157
FC whole brain, mean (SD)	1.08 (0.07)	1.11 (0.06)	1.07 (0.06)	1.08 (0.08)	*P* = 0.119

*P*-values were calculated from one-way ANOVA tests between clinical phenotypes.

Significant tests are highlighted with *P*-values in bold.

N, number of participants; SD, standard deviation; IQR, interquartile range; BPF, brain parenchymal fraction; RNFL, retinal nerve fibre layer; FDC, fibre density and cross section; FD, fibre density; FC, fibre cross section; CIS, clinically isolated syndrome; RRMS, relapsing–remitting multiple sclerosis.

### Baseline correlations

At baseline, we found significant correlations between imaging measures, demographics and disease duration (see Table 2). BPF, RNFL thickness, FDC and FC showed a negative correlation with EDSS (*ρ* = −0.368, *ρ* = −0.408, *ρ* = −0.365 and *ρ* = −0.397, respectively). BPF and RNFL thickness correlated with disease duration. In addition, we found significant correlations between imaging measures. Specifically, whole-brain FDC, FD and FC correlated with BPF (*r* = 0.451, *r* = 0.349 and *r* = 0.378, respectively), lesion fraction (*r* = −0.436, *r* = −0.424 and *r* = −0.267, respectively) and RNFL thickness (*r* = 0.532, *r* = 0.346 and *r* = 0.481, respectively).

**Table 2:** Baseline correlations between imaging measures, demographics and disease severity

	Age	Sex	Disease duration	EDSS	FDC	FD	FC
BPF	**r = −0.567** ***P* < 0.001**	r = −0.045 *P* = 0.734	**r = −0.398** ***P* = 0.002**	**ρ = −0.368** ***P* = 0.006**	**r = 0.451** ***P* < 0.001**	**r = 0.349** ***P* = 0.007**	**r = 0.378** ***P* = 0.003**
Lesion fraction	**r = 0.272** ***P* = 0.037**	r = 0.176 *P* = 0.183	r = 0.223 *P* = 0.076	ρ = 0.220 *P* = 0.107	**r = −0.436** ***P* = 0.001**	**r = −0.424** ***P* = 0.001**	**r = −0.267** ***P* = 0.041**
RNFL thickness	**r = −0.429** ***P* = 0.001**	r = 0.163 *P* = 0.226	**r = −0.384** ***P* = 0.004**	**ρ = −0.514** ***P* < 0.001**	**r = 0.557** ***P* < 0.001**	**r = 0.455** ***P* < 0.001**	**r = 0.528** ***P* < 0.001**
FDC	**r = −0.313** ***P* = 0.016**	r = 0.097 *P* = 0.463	r = −0.069 *P* = 0.605	**ρ = −0.365** ***P* = 0.006**	**—**	**—**	**—**
FD	**r = −0.312** ***P* = 0.016**	**r = −0.320** ***P* = 0.014**	r = −0.064 *P* = 0.628	ρ = −0.227 *P* = 0.096	**—**	**—**	**—**
FC	r = −0.246 *P* = 0.061	**r = 0.355** ***P* = 0.006**	r = −0.052 *P* = 0.694	**ρ = −0.397** ***P* = 0.003**	**—**	**—**	**—**

Significant tests are highlighted with *P*-values in bold.

***Abbreviations:*** BPF, brain parenchymal fraction; RNFL, retinal nerve fibre layer; FDC, fibre density and cross section; FD, fibre density; FC, fibre cross section; EDSS; expanded disability status scale.

### Annual change comparisons: all participants and between multiple sclerosis phenotypes

**Figure 1:**
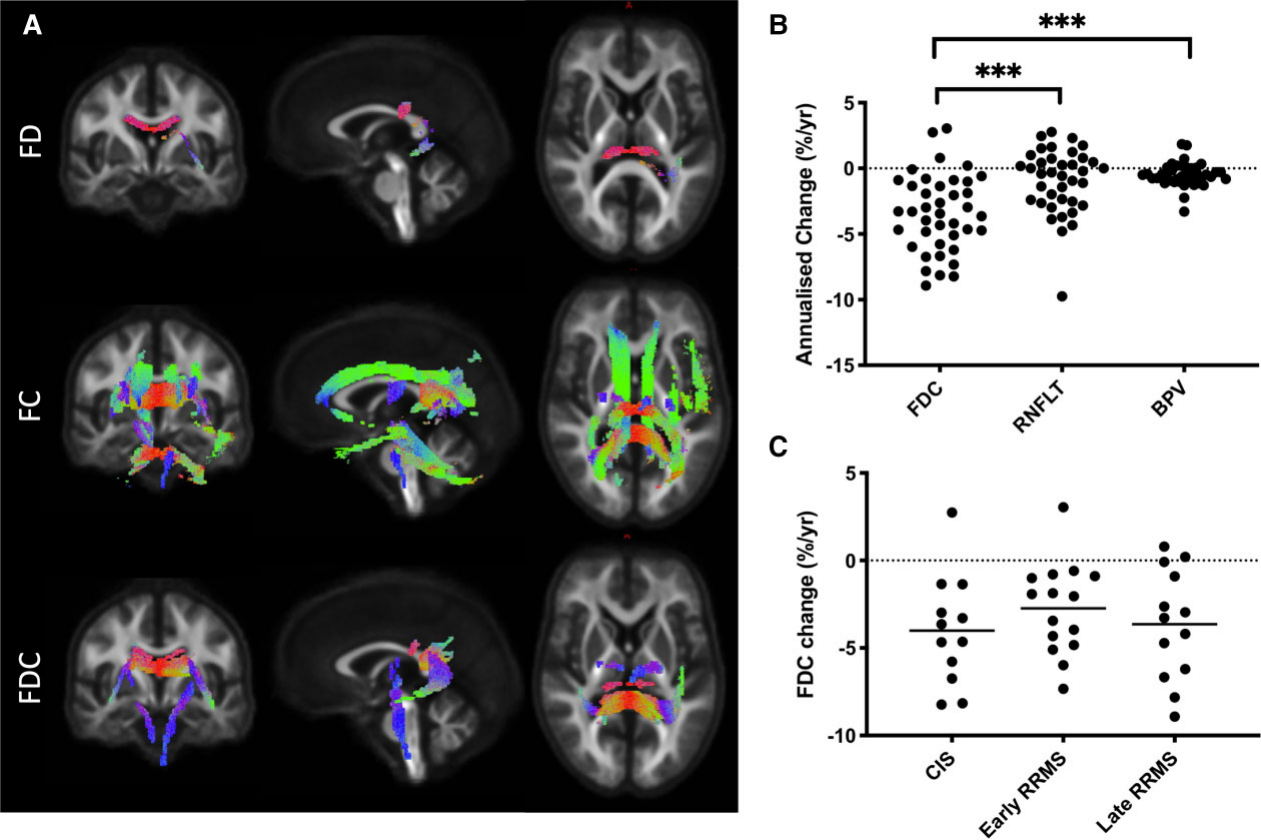
**Longitudinal change in fibre-specific measures in white matter tracts.** (**A**) White matter fibre tracts exhibiting significant longitudinal change in fibre-specific measures (FWER_corr_*P* < 0.05). The colour of the tracts indicates the local fibre orientation (green: dorsal-ventral, red: left-right, blue: cranial-caudal). The greatest change was observed for FC indicating tract atrophy. (**B**) The magnitude of change in FDC was on average over four and seven times greater than concomitant RNFL thinning (*P* < 0.0001) and whole-brain atrophy (*P* < 0.0001) respectively (mixed-effects GLM with group pair-wise *post hoc* tests). (**C**) There was no difference in the degree of change in FDC between multiple sclerosis sub-groups (one-way ANOVA). yr, year; FDC, fibre density and cross section; FD, fibre density; FC, fibre cross section; RNFLT, retinal nerve fibre layer thickness; BPV, brain parenchymal volume; CIS, clinically isolated syndrome; RRMS, relapsing–remitting multiple sclerosis.

Significant longitudinal change in fibre-specific measures (FD, FC and FDC) was observed in multiple white matter pathways including the cingulum, cortico-spinal tracts and corpus callosum (Fig. 1A). Annualized change in fibre-specific measures from these regions for all patients and the phenotypic sub-groups are reported in Table 3. The average annualized changes for fibre-specific markers were as follows: FDC −3.49 ± 3.32% p/a (*P* < 0.001); FD −2.39 ± −2.28% p/a (*P* < 0.001) and FC −0.99 ± 1.38% p/a (*P* < 0.001). BPV (−0.53 ± 0.95% p/a, *P* < 0.001), RNFL thickness (−0.75 ± 2.50% p/a, *P* = 0.036) and lesion fraction (6.90 ± 18.6% p/a, *P* = 0.009) also changed over time.

**Table 3:** Percentage annualized change for all imaging measures

	All patients	*P* ^a^	CIS	Early RRMS	Late RRMS	*P* ^b^
ΔBPF	−0.53 (0.95)	***P* < 0.001**	−0.46 (1.16)	−0.78 (0.97)	−0.29 (0.64)	*P* = 0.263
ΔLesion fraction	6.90 (18.6)	***P* = 0.009**	8.83 (17.53)	4.51 (24.14)	8.30 (9.99)	*P* = 0.741
ΔRNFL thickness	−0.82 (2.17)	***P* = 0.025**	−0.48 (2.31)	−1.38 (2.66)	−0.51 (1.24)	*P* = 0.493
ΔFDC	−3.49 (3.32)	***P* < 0.001**	−4.88 (3.71)	−2.47 (3.06)	−3.56 (2.99)	*P* = 0.093
ΔFD	−2.39 (2.28)	***P* < 0.001**	−3.46 (2.79)	−1.48 (1.74)	−2.72 (2.07)	***P* = 0.029**
ΔFC	−0.99 (1.38)	***P* < 0.001**	−1.14 (1.25)	−1.00 (1.62)	−0.84 (1.20)	*P* = 0.843

Significant tests are highlighted with *P*-values in bold.

BPF, brain parenchymal fraction; RNFL, retinal nerve fibre layer; FDC, fibre density and cross section; FD, fibre density; FC, fibre cross section; CIS, clinically isolated syndrome; RRMS, relapsing–remitting multiple sclerosis.

^a^ One-sample *t*-test.

^b^ one-way ANOVA.

Focusing further on FDC as a composite microstructural and macrostructural marker of axonal loss, ANOVA showed significant differences in the rates of change in neurodegenerative markers (*P* < 0.05) with Tukey *post hoc* tests revealing significantly more rapid decline in FDC compared with BPV (*P* < 0.0001) and RNFL thickness (*P* < 0.0001), yet no difference between BPV and RNFL thickness (Fig. 1B). The rate of change in FDC did not differ between clinical sub-groups (Fig. 1C).

### Annual change correlations

Table 4 shows the correlation between annual change measures. A significant correlation was found between an annual change in BPF and FDC (*r* = 0.459, *P* < 0.001), FD (*r* = 0.312, *P* = 0.026) and FC (*r* = 0.577, *P* < 0.001). An annual change in lesion fraction was associated with a change in FC (*r* = 0.377, *P* = 0.005).

**Table 4:** Correlations between annualized change in imaging measures

	ΔFDC	ΔFD	ΔFC
ΔBPF	**r = 0.459** ***P* < 0.001**	**r = 0.312** ***P* = 0.026**	**r = 0.577** ***P* < 0.001**
Δlesion fraction	r = 0.186 *P* = 0.178	r = 0.125 *P* = 0.383	**r = 0.377** ***P* = 0.005**
ΔRNFL	r = 0.028 *P* = 0.865	r = 0.132 *P* = 0.431	r = 0.060 *P* = 0.721

Significant tests are highlighted with *P*-values in bold.

BPF, brain parenchymal fraction; RNFL, retinal nerve fibre layer; FDC, fibre density and cross section; FD, fibre density; FC, fibre cross section

### Differences between >/<−0.4% per annum brain volume change

We did not find any differences in the baseline characteristics between patients with >/<−0.4% brain volume change (Supplementary Table 1). However, the group with <−0.4% annualized brain volume change had a greater annual loss of FC (*P* = 0.009) compared with the group with >−0.4% annual brain volume change (Supplementary Table 2). Patients with >−0.4% brain volume change exhibited loss of FD (*P* < 0.001) and FDC (*P* = 0.003).

## Discussion

Prevention of disability progression is currently the most important goal for the clinical treatment and management of pwMS. Axonal degeneration is considered to be a major cause of irreversible neurological disease progression;^1,2^ however, there are no techniques capable of measuring neuro-axonal degeneration over short, routine clinical follow-up times. This study aimed to determine the sensitivity of fibre-specific measures as a marker of change within clinical follow-up periods relative to other putative neurodegenerative markers (brain volume and RNFL thickness) in patients with varying disease durations. We found that the rate of change in fibre-specific measures was more rapid than the rate of change in brain volume or RNFL thickness and did not differ between patients with and without ‘clinically meaningful’ brain atrophy (<−0.4% per annum).

Current high-efficacy disease-modifying therapies have been shown to be able to stabilize the disease course for people with RRMS. This results in difficulties for measuring progression over short periods and/or an increased need for longer follow-up studies. In this study, a change in EDSS over a 1-year period was only seen in two of 40 patients followed up longitudinally. Furthermore, the average annual brain atrophy was 0.5% with a standard deviation of 0.95%. This included a number of patients with increased brain volume. In comparison, the fibre-specific measures showed an annual loss of 3.46%, which is almost seven times larger than brain atrophy measures. Only two patients showed an increase in fibre-specific measures, both of which showed significant oedema due to newly appearing acute inflammation (based on visual observation). The latest NEDA criteria (NEDA-4) includes an additional criterion of no brain volume change of <−0.4%.^6^ Interestingly, we found that patients with sub-threshold brain volume loss exhibited significant loss of FD and FDC indicative of progressive axonal loss. Finally, we found that both brain volume and fibre-specific measures correlated similarly with disease severity. Taken together, these findings support the proposal that fibre-specific measures of axonal degeneration are more sensitive to structural change, especially over a 1-year period, highlighting their potential in monitoring disease progression in pwMS.

Another key finding in the current study is the rates of annualized change in fibre-specific measures of axonal degeneration between the different stages of multiple sclerosis. People with CIS showed a greater reduction in FD over a 1-year period compared with people with late RRMS. In a cross-sectional study examining early multiple sclerosis patients with optic neuritis, the authors found that FD was greatly reduced compared with FC within the optic radiations.^16^ In addition, Storelli *et al.*^25^ found more pronounced changes in whole-brain FC in progressive multiple sclerosis compared with RRMS. Together with the present findings, these data indicate that microscopic changes in axonal density and macroscopic changes in white matter tract cross section do not occur at the same rate throughout the disease course. Therefore, early in the disease course, a reduction of FD might dominate any measurable changes seen in FC, whereas changes in FC might become more pronounced later in the disease, resulting in more large-scale atrophy. Therefore, the proposed use of volumetric information for estimating disease trajectories could be less sensitive at early disease stages. Unfortunately, there are a very limited number of studies employing fibre-specific analysis in progressive stages of multiple sclerosis, and no previous studies have assessed longitudinal rates of axonal loss in patients across the disease spectrum. Larger longitudinal studies will be needed to ascertain more precisely the variation in trajectories of axonal loss across the disease and patients and the relative timings of change in axonal density and cross section (atrophy) over the whole course of multiple sclerosis.

We also examined the use of RNFL thickness as a measure of neuro-axonal degeneration in multiple sclerosis. We found RNFL thinning of 0.75% per year, which in our sample equates to −0.73 μm per year. Previous studies evaluating the annual change of RNFL have reported average thinning between −0.36 and −1.49 μm per year,^26–29^ with one study not finding any significant changes for more than 2 years.^30^ This inconsistency could be due to the sample of patients recruited and the highly specific nature of the measurement, i.e. pwMS without damage to their visual system might show no RNFL thinning, whereas pwMS with severe optic neuritis might have an apparent RNFL thinning. Compared with annual brain atrophy, the annual change of RNFL thickness is 1.5 times larger over a 1-year period. However, compared with the annual change of fibre-specific measures, the annual change of RNFL is four times smaller with several patients displaying an apparent increase in RNFL thickness suggesting measurement inaccuracy. Together, these findings show potential for the use of RNFL thickness as a biomarker for progression in multiple sclerosis; however, fibre-specific measurements might provide a more sensitive marker.

### Limitations and future directions

This study has several limitations. First, the study was a retrospective design using a convenience sample of patients who were scanned with the relevant dMRI protocol. Thus, the sample was not large, included no patients with progressive multiple sclerosis phenotypes and contained missing data and non-standardized follow-up durations. Nonetheless, the present study is a useful proof-of-concept for demonstrating effect sizes that can be used for planning future larger prospective studies. Second, we detected no to little longitudinal change in EDSS, making it challenging to investigate the clinical relevance of progressive axonal loss. More sensitive functional measures of disability progression and/or more long-term longitudinal studies will be required to validate the use of fibre-specific markers as predictive of functional decline. Third, OCT data in this study were limited by the inclusion of data from two separate scanners and the lack of additional measures beyond RNFL thickness. This reflects the limitations of collecting data during clinical monitoring rather than prospectively planned data collection. However, individual patients were scanned on the same scanner over time, and we could not find evidence for gross differences in sensitivity between the scanners in the literature. In addition, the data were collected before the publication of important standards for OCT quality assurance^31^ and reporting;^32^ therefore, the data were not acquired with these factors in mind. Finally, brain volumetric measures employed in this study were restricted to whole-brain rather than grey and white matter volumes separately due to the use of FLAIR for brain volume calculation and inconsistent collection of 3D T_1_-weighted imaging. It is possible that greater sensitivity to atrophy can be obtained by focusing on specific tissue types or even specific brain structures. However, to date, there are no sub-structure volumetric measures that have been validated for clinical use and the reproducibility of brain volume measures appears to be highest for total brain volume. We found that whole-brain volumes calculated using FLAIR were strongly correlated with volumes calculated from 3D T_1_-weighted scans and therefore can provide useful volumetric data for cohorts where T_1_-weighted images are unavailable. Future studies should investigate relationships between longitudinal change in fibre-specific measures and segmental brain volume measures.

We contend that there is scope for improving radiological monitoring of multiple sclerosis and techniques such as fixel-based analysis could contribute. A recent review article highlighted some of the hurdles facing clinical translation of this technology but perhaps most important is the need for the integration of analysis pipelines into clinical workflows. This is already occurring for volumetric analysis (e.g. SIEMENS&rsquo; ‘MorphoBox’ volumetric analysis toolkit). Given the sensitivity of advanced diffusion analysis could follow suit in this way with assistance from vendors or as an alternative model, via third-party companies providing timely analysis support. Initially, we think this technology is more likely to be used in early phase trials as a gateway towards use in standard clinical monitoring. Another potential use of this technology could be in developing composite biomarkers including advanced diffusion, OCT and serum biomarkers.^33,34^ Future studies are required to study the covariance and timing of change for brain, ocular and serum markers of neuro-axonal loss.

The reproducibility of the technology is high if the MRI protocol remains unchanged (this is based on our in-house unpublished data in healthy individuals). This is an obvious issue for use in clinical radiological settings. However, most quantitative MRI methods, including volumetric analyses, suffer from this same limitation.

## Conclusions

This is the first study to examine the longitudinal sensitivity of fibre-specific measures in multiple sclerosis. We found that fibre-specific measures can be derived from data collected within a standard clinical radiological multiple sclerosis study and are considerably more sensitive to longitudinal change than whole-brain atrophy and RNFL thinning. These results show that fibre-specific measures offer a potential means to clinically monitor neuro-axonal degeneration in pwMS or as early-stage clinical trial end-points for neuroprotective therapies. Larger studies, including more progressive stages of multiple sclerosis, are now required to establish the use of fibre-specific measures in these contexts.

## Supplementary Material

fcac065_Supplementary_Data

## Abbreviations

BPF =: brain parenchymal fraction
BPV =: brain parenchymal volume
CIS =: clinically isolated syndrome
dMRI =: diffusion MRI
FC =: fibre cross section
FD =: fibre density
FDC =: fibre density and cross section
FLAIR =: fluid-attenuated inversion recovery
FOD =: fibre orientation distribution
GLM =: general linear model
NEDA =: no evidence of disease activity
OCT =: optical coherence tomography
RNFL =: retinal nerve fibre layer
RRMS =: relapsing–remitting multiple sclerosis
