# Supplementary material for: Longitudinal tracking of axonal loss using diffusion magnetic resonance imaging in multiple sclerosis
Source: Brain Commun. 2022 Mar 17;4(2):fcac065. doi: 10.1093/braincomms/fcac065 (PMC9006042; doi:10.1093/braincomms/fcac065)
Supplement: fcac065_Supplementary_Data [file fcac065_supplementary_data.docx]

**Supplementary Figures**


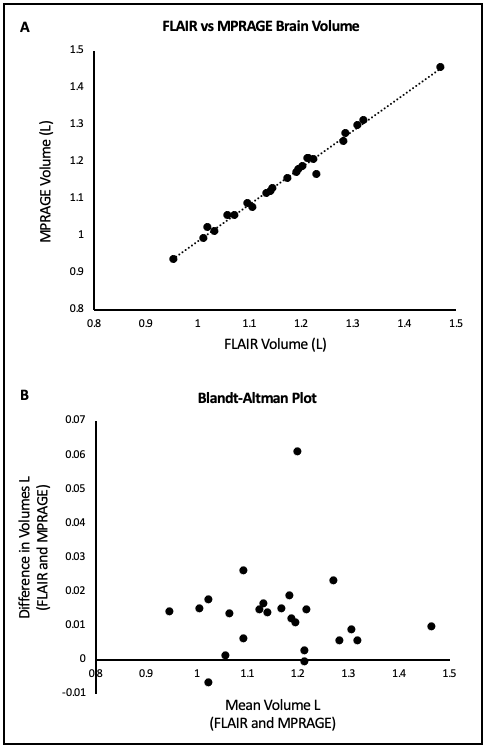


***Supplementary Figure 1. (A)*** Scatterplot showing the strong relationship between FLAIR and MPRAGE derived brain volumes. ***(B)*** Blandt-Altman Plot of the FLAIR and MPRAGE derived brain volumes showed that volumes were slightly larger when calculated using FLAIR, yet the mean difference was small compared to the mean volume (<1%).


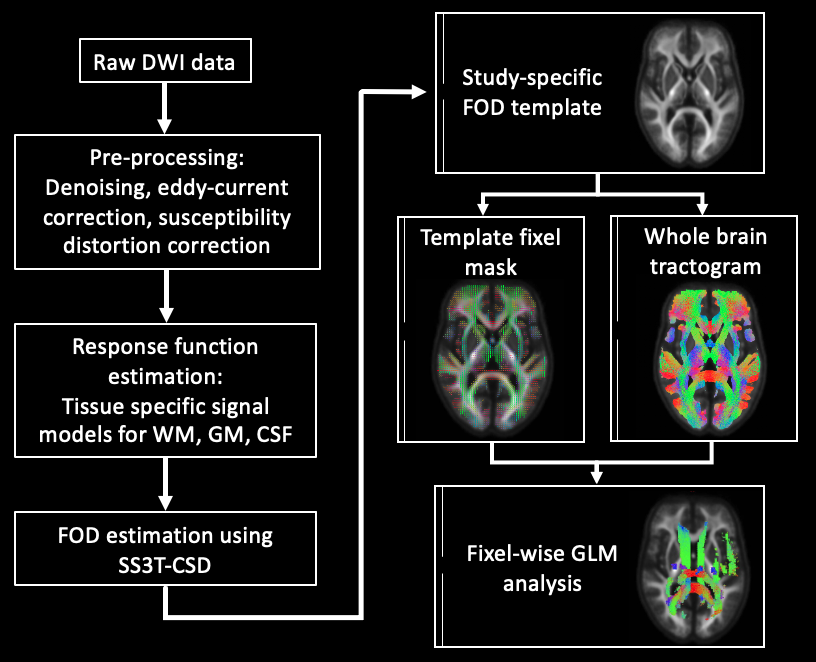


***Supplementary Figure 2.*** Diagrammatical overview of analysis of the diffusion MRI.

***Abbreviations:*** DWI, diffusion weighted imaging; WM, white matter; GM, grey matter; CSF, cerebral spinal fluid; FOD, fibre orientation distribution; SS3T-CSD, single-shell 3-tissue constrained spherical deconvolution; GLM, general linear model.

**Supplementary Tables**

***Supplementary Table 1.*** Differences in baseline variables between patients with >/< -0.4% brain volume change. Unless otherwise stated, values reflect mean (SD).

|  | **∆BPV > -0.4%** | **∆BPV <= -0.4%** | **p** |
| --- | --- | --- | --- |
| **N** | 35 | 24 |  |
| **Age (years)** | 42.04 (11.69) | 38.56 (9.52) | p=0.233 |
| **Sex (female %)** | 88.6% | 83.3% | p=0.564 |
| **DD (years)** | 6.47 (7.71) | 5.85 (7.30) | p=0.757 |
| **EDSS, median (IQR)** | 0 (0,1) | 0 (0,1) | p=0.259 |
| **BPF (%)** | 84.5 (4.91) | 85.8 (4.59) | p=0.311 |
| **Lesion fraction (% BPV)** | 0.39 (0.38) | 0.63 (0.68) | p=0.089 |
| **RNFL (**μ**m)** | 95.28 (14.46) | 99.27 (10.07) | p=0.264 |
| **whole brain FDC** | 0.35 (0.03) | 0.36 (0.03) | p=0.343 |
| **whole brain FD** | 0.32 (0.01) | 0.33 (0.02) | p=0.425 |
| **whole brain FC** | 1.08 (0.07) | 1.09 (0.07) | p=0.377 |

***Supplementary Table 2.*** Differences in longitudinal change in paraclinical variables between patients with >/< -0.4% brain volume change.

|  | **∆BPV > -0.4%** | **∆BPV <= -0.4%** | **p** |
| --- | --- | --- | --- |
| **∆FDC (%)** | -2.49 (3.54) | -4.17 (3.03) | p=0.054 |
| **∆FD (%)** | -2.1 (2.25) | -2.6 (2.33) | p=0.314 |
| **∆FC (%)** | -0.36 (1.30) | -1.42 (1.27) | **p=0.010** |
| **∆lesion fraction (%)** | 10.11 (18.67) | 4.7 (18.53) | p=0.204 |
| **∆RNFL Thickness (%)** | -0.54 (2.93) | -0.92 (2.15) | p=0.981 |
